# PBMC and fibroblast cocultures to mimic the *in vivo* effect of BCG on trained immunity

**DOI:** 10.17912/micropub.biology.001449

**Published:** 2025-02-05

**Authors:** David Merriam, Adriana Weinberg

**Affiliations:** 1 Department of Pediatrics, University of Colorado Anschutz Medical Campus, Aurora, United States; 2 Department of Biology, Metropolitan State University of Denver, Denver, Colorado, United States; 3 Departments of Pediatrics, Medicine, and Pathology, University of Colorado Anschutz Medical Campus, Aurora, Colorado, United States

## Abstract

BCG greatly stimulates innate immune cells. Previous studies demonstrated that BCG-stimulated monocytes develop trained immunity whereby they respond to homologous and heterologous antigens. Previous studies used isolated monocytes or animal models to study BCG-induced trained immunity, which have benefits and limitations. To approximate *in vivo* conditions, we stimulated peripheral blood mononuclear cells (PBMCs) with BCG-treated human fibroblasts. We found that compared with BCG stimulation, the addition of fibroblasts increased the expression of IFN-γ in NK and γδ T cells and of TNF-α and IL-10 in monocytes. We conclude that BCG-treated fibroblasts offer advantages over BCG alone for studying trained immunity.

**Figure 1. PBMC and Fibroblast responses to BCG stimulation:**
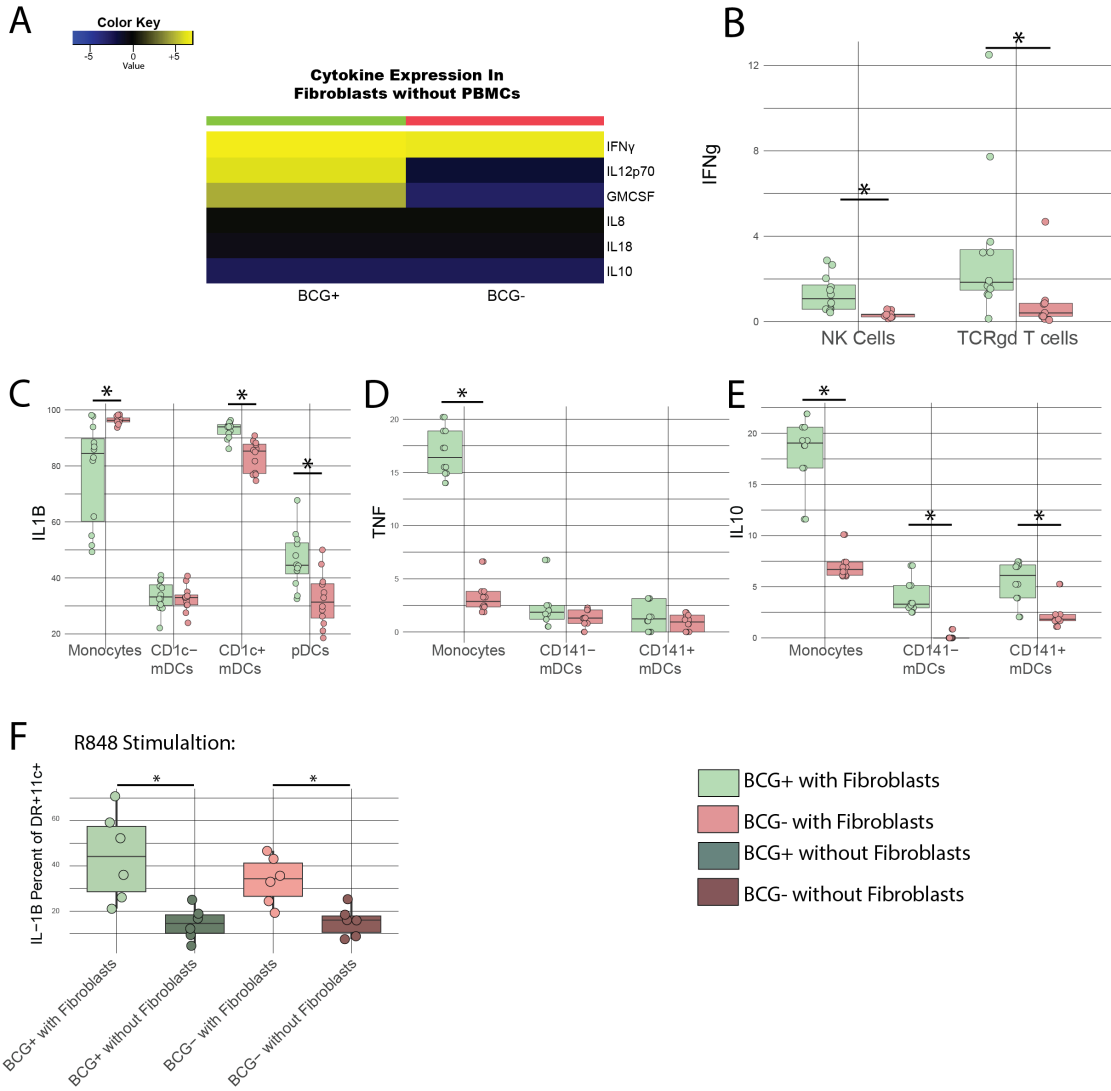
Fibroblasts were stimulated with BCG overnight prior to addition of PBMCs and compared to unstimulated Fibroblasts. A) Cytokine expression in Fibroblasts the following day prior to addition of PBMCs. PBMCs were added to stimulated fibroblasts and compared via flow cytometry to unstimulated controls. B) Expression of IFN-γ on NK Cells or 482 TCRγδ cells in response to BCG stimulation of fibroblasts. C-E) Expression of C)IL-1β, D)TNF, or E)IL-10 on Monocytes or myeloid Dendritic Cell subsets. F) PBMCs were then rested for 3 days and restimulated with R848, and myeloid cells were assessed for expression of IL-1β and compared with BCG unstimulated controls or BCG stimulated without fibroblast controls.

## Description

Bacille Calmette-Guerin (BCG) is a vaccine against *Mycobacterium tuberculosis* that may also confer nonspecific protection against heterologous infections in adults (Arts et al., 2018; Giamarellos-Bourboulis et al., 2020; Jensen et al., 2015; Netea et al., 2021) and all-cause infectious morbidity and mortality in infants (Curtis, 2019) . The mechanism of the putative nonspecific protection conferred by BCG has been ascribed to the development of trained immunity, defined as the acquisition of immunologic memory by innate immune cells (Brueggeman et al., 2022; Kleinnijenhuis, Quintin, Preijers, Benn, et al., 2014) .

*Ex vivo* treatment of monocytes isolated from peripheral blood mononuclear cells (PBMCs) with BCG was previously shown to increase expression of monocyte-derived IFNγ, TNFα, and IL-1β (Kleinnijenhuis et al., 2012) . BCG vaccination in humans was associated with activation of Th1, Th17, γδ T cells, and Natural Killer (NK) cells (Kleinnijenhuis, Quintin, Preijers, Benn, et al., 2014; Kleinnijenhuis, Quintin, Preijers, Joosten, et al., 2014). BCG possesses a number of known pattern recognition receptor agonists (Dowling et al., 2017; Means et al., 1999; Yadav & Schorey, 2006) , which stimulate antigen presenting cells (APCs) to activate T cell responses through multiple pathways including cytokine secretion such as TNF-α, IL-12, IL-1β, and IL-6. (Moliva et al., 2017)

Current models of trained immunity frequently rely on lymphocyte deficient animal models or pure myeloid cultures to avoid effects of conventional immunological memory that may skew results (Bekkering et al., 2016; Kleinnijenhuis et al., 2012; Kleinnijenhuis, Quintin, Preijers, Benn, et al., 2014). However, these models omit critical immune cells or key interactions between the immune system and other tissues.

To address this gap, we sought to develop an *in vitro* model of the effect of BCG on monocytes and other innate immune cells that includes a complex system similar to *in vivo* environments, including adaptive immune cells and cells present in the subcutaneous tissue where BCG is inoculated. Here we incubate fibroblasts with BCG, after which we add PBMCs to the culture. Fibroblasts have multiple TLRs and are likely to respond to BCG stimulation (Bekkering et al., 2016) .

To determine if BCG and fibroblasts interacted ex vivo, we compared the cytokine secretion of BCG-exposed and unexposed fibroblasts. We found detectable levels of IFN-g, IL-12, IL-8, IL-18, IL-10, and GM-CSF in culture supernatants of both exposed and unexposed fibroblasts. However, BCG-exposed fibroblasts secreted higher levels of IFN-g, IL-12, and GM-CSF compared with unexposed fibroblasts (Fig A), indicating that the fibroblasts responded to BCG infection.

Next, we compared the functional and phenotypic characteristics of PBMCs exposed to BCG-treated or untreated fibroblasts. BCG stimulation of fibroblasts drove increased expression of IFN-γ on both NK cells and γδ T cells (Fig B); increased the expression of IL-1β in CD1c+ cDCs and pDCs (Fig C); and increased IL-10 expression in CD141+ and CD141- cDCs (Fig E). BCG-treated fibroblasts also decreased expression of IL-1β on monocytes while increasing TNF-α and IL-10 expression compared to untreated fibroblasts (Fig C-E).

We then stimulated PBMCs for 3 days in the presence of BCG-treated fibroblasts, BCG without fibroblasts or unstimulated control. After 3 days of stimulation no fibroblasts were visible under microscopy. We harvested, counted, rinsed, and rested these PBMCs for 3 additional days and stimulated them with R848, a potent TLR 7 and 8 agonist capable of eliciting immune stimulation at concentrations as low as 0.1μg/mL (Ahonen et al., 1999) . PBMCs stimulated with BCG in the presence of fibroblasts had reduced viability compared to PBMCs stimulated without fibroblasts or unstimulated controls. Because there were insufficient monocytes, pDCs or mDCs to effectively compare the effect of treatment on each subset, we assessed cytokine production in response to R848 in all myeloid cells, defined as CD3- CD56- CD19- HLA-DR ^HI^ CD11c+. Myeloid cells cultured in the presence of BCG-treated fibroblasts showed higher expression of IL1-β upon R848 restimulation than PBMCs stimulated with BCG in the absence of fibroblasts or control unstimulated PBMC (Fig F).

*In vitro* induction of trained immunity has been witnessed previously using monocytes alone to avoid confounding effects from other leukocytes present in peripheral blood (Arts et al., 2015; Madura Larsen et al., 2007) , but more recent studies have complemented this work identifying similar effects on PBMCs from vaccinated adults (Tarancón et al., 2020) or from training PBMCs and assessing responses against heterologous stimuli (Kilic, Debisarun, et al., 2024; Kilic, Matzaraki, et al., 2024) . By including fibroblasts with PBMCs, our work provides a novel cell-culture tool to study immune functions closer to *in-vivo* conditions.

Further development of this assay will improve survival of cells after training and induce a more physiologically relevant training response. While fibroblasts were nonviable after 3 days of stimulation with BCG and PBMCs, residual BCG-infected fibroblasts may have persisted; future efforts to refine this assay should consider heat-killed BCG as a control. Our results suggest a shorter in vitro training period than 72 hours may be beneficial; previous studies were able to induce the trained immunity phenotype in monocytes after as little as 24 hours of stimulation (Angelidou et al., 2018; Arts et al., 2015; Buffen et al., 2014) . The ratio of BCG cfu/leukocyte of 0.02 in our study was within the 0.005 to 1 cfu/cell range used in previous studies (Buffen et al., 2014; Madura Larsen et al., 2007) , but assessing multiple concentrations should be considered for future studies, not just in terms of cfu per leukocyte but also cfu per fibroblast. Increasing the resting time after trained immunity, perhaps in conjunction with a shorter stimulation time, may also result in reduced cell death and increased memory. Previous studies have shown signs of trained immunity as early as 3 days of resting but up to a week was sometimes required (Bekkering et al., 2016) . Due to the loss of cell viability, we were unable to incubate cells in our experiment longer. There is marked heterogeneity between the different strains of BCG used by vaccine manufacturers (Angelidou et al., 2020) , which should be considered when attempting to replicate results. We used strain Pasteur, compared to other research which used strains Danish 1331 (Arts et al., 2015; Madura Larsen et al., 2007) or Bulgaria (Namakula et al., 2020) . Overall, the induction of trained immunity using BCG *in vitro* stimulation seems to be very sensitive to small differences in experimental conditions. For example, in a previous study, high concentrations of BCG resulted in loss of training-induced secondary responses seen at lower doses and was also associated with a relative loss of macrophage viability (Angelidou et al., 2018) .

Human subjects were healthy volunteers who had provided blood for platelet donations. Although healthy, it is possible that some of these volunteers were exposed to another agent capable of inducing trained immunity. Non-tuberculosis *Mycobacteria* (NTM) are prevalent in municipal water supplies in the United States (Gebert et al., 2018) and while limited immunological studies exist to study the effects of trained immunity, some strains have demonstrated the ability to activate TLR2 and dectin-1, similar to BCG (Shah et al., 2019) . Proper controls should catch such outliers.

The fibroblasts responded to BCG exposure with enhanced production of cytokines. In addition, in our trained immunity experiments, the presence of fibroblasts in the experimental model enhanced production of cytokines and their presence increased inflammatory responses to restimulation in myeloid APCs. Thus, the inclusion of fibroblasts in *in vitro* models of BCG-induced trained immunity warrants further studies.

## Methods

**Study population**

The study consisted of in vitro experiments included in a human subject study approved on 27 May 2020 by the Colorado Multiple Institution Research Board (protocol COMIRB 20-0593). Human samples for this study used archived deidentified viably cryopreserved PBMCs that would normally be discarded from anonymous healthy adult platelet donations (HANC, 2011) which used citrate as an anticoagulant. On thaw, viability was confirmed with Guava Viacount [Cytek] per the manufacturer’s instructions. Informed consent for this portion of the study was not required as all information was deidentified.

**BCG Stimulation Assays**

Fetal lung fibroblasts were cultured with DMEM + 20% FBS for 1-2 days prior to the assay in 24-well plates [Corning], until confluent. Cultures were rinsed and provided fresh media prior to addition of BCG.

BCG substrain Pasteur was diluted in serum free AIM-V medium [Gibco] and added to fibroblasts at a concentration of 4*10 ^4^ cfu/mL. 24 hours later, we aspirated media and rinsed all wells in PBS. Thawed 2*10 ^6^ PBMCs were resuspended in 200ul AIM-V and added to each well, then incubated for 3 days at 37°C with 5% CO _2_ . Suspended PBMCs were collected and adherent PBMC were removed using Accutase™ [Thermofisher], pooled with suspended cells, rinsed in PBS, and counted using Guava easyCyte™ [Millipore] and ViaCount™ staining [Millipore]. A subset of these treated PBMCs were then rinsed and placed in fresh AIM-V media to rest for 3 additional days. Next, the cells were restimulated overnight with 500ul of 0.1 µg/mL of R848 [Mabtech] with 5µg/ml Brefeldin A [Sigma] and fresh AIM-V media. Cells were washed and stained for flow cytometry.

**Flow Cytometry**

All incubation steps were performed at 4˚C in the dark and followed standardized flow cytometry principles (Maecker et al., 2005) . Anti-CD107a and 5µg/mL of Monensin [BD Biosciences] were added 5 hours prior to the assay if included in the panel. In all panels including CD14 staining, cells were treated with TrueStain FcX™ [Biolegend] prior to surface staining, and True-stain monocyte blocker™ [Biolegend] was included in all staining solutions. Cells were incubated in BD FACS lysing solution [BD] and BD Perm Buffer II [BD] before intracellular staining. Cells were captured on the Quanteon™ cytometer (Novocyte) within 3 hours of staining.

**Detection of cytokines in supernatant**

Supernatant from initial assays were diluted 1:100 or 1:1000-fold established during assay optimization and tested for the following cytokines and chemokines using Meso Scale Discovery chemiluminescence microarrays U-plex™ kits following the manufacturer’s instructions: IL-12 (p70), IL-18, IL-10, IFN-γ, GM-CSF, IL-8, IFN-α2a, IL-1β, TNF-α, IP-10, MCP-1, MIP-1α, MIP-1β, and IL-6.

**Statistical Analysis**

All statistical analyses were conducted using R programming language. Significance, defined by p value <0.05, was determined by Mann-Whitney U test.

## Reagents

**Table:** 

**Marker**	**Conjugated Fluorophore**	**Clone**	**Company**
**Panel 1**			
CD107a	PE	H4A3	BD Biosciences
TCRγδ	FITC	11F2	BD Biosciences
CD19	PerCP-Cy5.5	H1B19	Biolegend
CD14	PerCP-Cy5.5	MφP9	BD Biosciences
CD56	PE-CF594	B159	BD Biosciences
CD16	PE-Cy7	3G8	BD Biosciences
CD8	APC-Cy7	SK1	BD Biosciences
CD3	Alexa Fluor 700	SP34-2	BD Biosciences
HLA-DR	APC	Tu36	Biolegend
IFN-γ	BV421	4S.B3	Biolegend
			
**Panel 2**			
PD-L1	BV421	MIH1	BD Biosciences
CD14	FITC	M5E2	BD Biosciences
CD123	PE-CF594	7G3	BD Biosciences
CD3	PerCP-Cy5.5	UCHT1	BD Biosciences
CD19	PerCP-Cy5.5	H1B19	Biolegend
CD56	PerCP-Cy5.5	B159	BD Biosciences
CD11c	PE-Cy7	3.9	Biolegend
CD1c	Alexa Fluor 700	L161	Biolegend
HLA-DR	APC-H7	G46-6	BD Biosciences
IL-1β	PE	AS10	BD Biosciences
IL-6	APC	MQ2-13A5	BD Biosciences
			
**Panel 3**			
CD14	FITC	M5E2	BD Biosciences
CD141	PE	M80	Biolegend
CD3	PerCP-Cy5.5	UCHT1	BD Biosciences
CD19	PerCP-Cy5.5	H1B19	Biolegend
CD56	PerCP-Cy5.5	B159	BD Biosciences
CD16	PE-Cy7	3G8	BD Biosciences
CD11c	Alexa Fluor 700	3.9	Biolegend
HLA-DR	APC-Cy7	G46-6	BD Biosciences
IL-10	BV421	JES3-9D7	BD Biosciences
TNF	PE-CF594	Mab11	BD Biosciences
IL-12	APC	C11.5	BD Biosciences
			
**Panel 4**			
CD14	FITC	M5E2	BD Biosciences
CD123	BV650	6H6	Biolegend
CD3	PerCP-Cy5.5	UCHT1	BD Biosciences
CD19	PerCP-Cy5.5	H1B19	Biolegend
CD56	PerCP-Cy5.5	B159	BD Biosciences
CD11c	PE-Cy7	3.9	Biolegend
CD1c	BV786	F10/21A3	BD Biosciences
HLA-DR	APC-H7	G46-6	BD Biosciences
IL-1β	PE	AS10	BD Biosciences
IL-6	APC	MQ2-13A5	BD Biosciences
IFN-γ	BV421	4S.B3	Biolegend
TNF	PE-CF594	Mab11	BD Biosciences

**Table 1. Flow cytometry monoclonal antibodies used in this study.**

**Table:** 

**Component**	**Company**	**Catalog Number**
Human Umbilical Vein Endothelial Cells (HUVECs)	Lonza	CC2519
T175 Flasks	Fisher	12-562-000
Endothelial Growth Medium 2	Lonza	CC3156 + CC-4176
	Includes FBS, Hydrocortisone, hFGF, VEGF, R3-IGF, Ascorbic Acid, hEGF, GA-1000, and Heparin	
DMEM	Gibco	11-965-092
FBS	GemCell	100-500
24-well tissue treated plates	Corning	07-200-84
AIM-V Media	Gibco	12055091
Ficoll-Hypaque	Sigma	H8889
Accutase	ThermoFisher	00-4555-56
R848	Mabtech	3611
Brefeldin A	Sigma	20350-15-6
Zombie Yellow	Biolegend	423103
TrueStain FcX	Biolegend	422301
True-stain Monocyte Blocker	Biolegend	426101
Paraformaldehyde	Electron Microscopy Sciences	15700
BD FACS Lysing solution	BD	349202
BD Perm Buffer II	BD	558052
Monensin	BD	554724
U-plex discovery kits	Meso Scale Discovery (MSD)	custom - see methods
CoolCell Freezing System	Corning	432004
Guava Viacount	Cytek	1835414

**Table 2. Reagents from manufacturers used in this study.**
